# Supplementary figures and images for: Foxi2 and Sox3 are master transcription regulators that control ectoderm germ layer specification in Xenopus
Source: PLoS Biol. 2025 Nov 4;23(11):e3003476. doi: 10.1371/journal.pbio.3003476 (PMC12599972; doi:10.1371/journal.pbio.3003476)

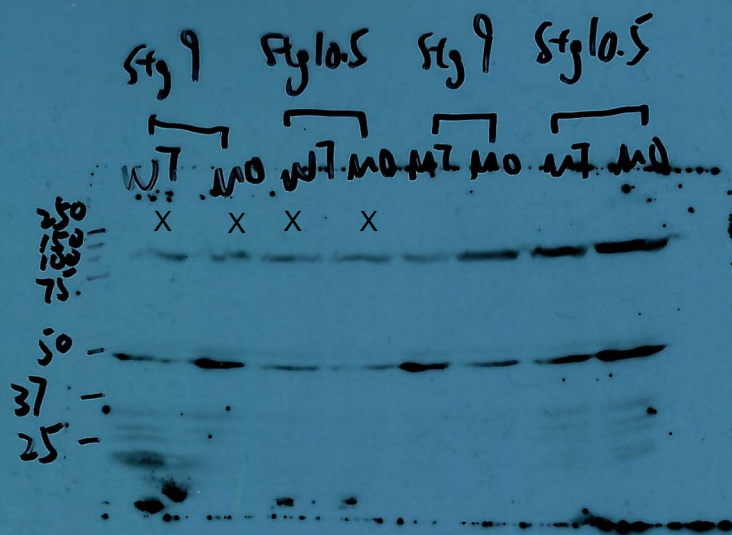

~~100~~

100eq.  
β-actin

1:2K β-actin mouse

1:40K mouse HRP

Ex 30min

SOX3

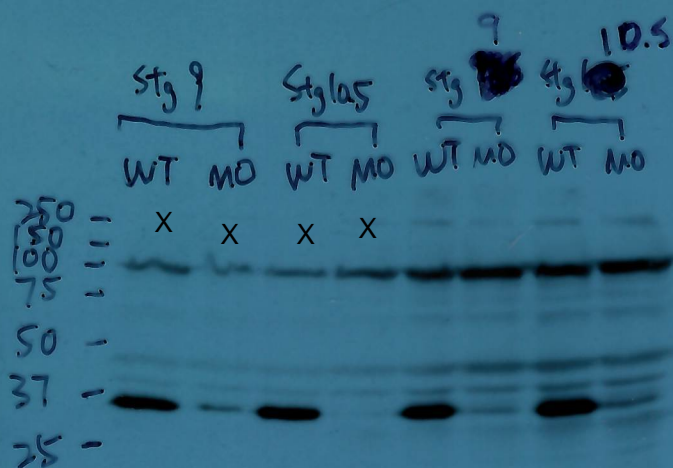

1:2K SOX3 rabbit

1:10K rabbit HRP

15s Exposure

100eq/lane

Supplement: S1 Raw Images — Validation of Foxi2 Protein Knockdown. Western blot was performed on Stage 9 and 10.5 embryos injected with Foxi2 morpholino at the 1–2 cell stage. Foxi2 (left) and alpha-Tubulin (right) expression are compared between wild-type and morpholino-treated embryos. The two raw exposures used for the Foxi2 and alpha-Tubulin bands in the main figure text are presented in the middle and bottom figures. (PDF) [file pbio.3003476.s019.pdf]

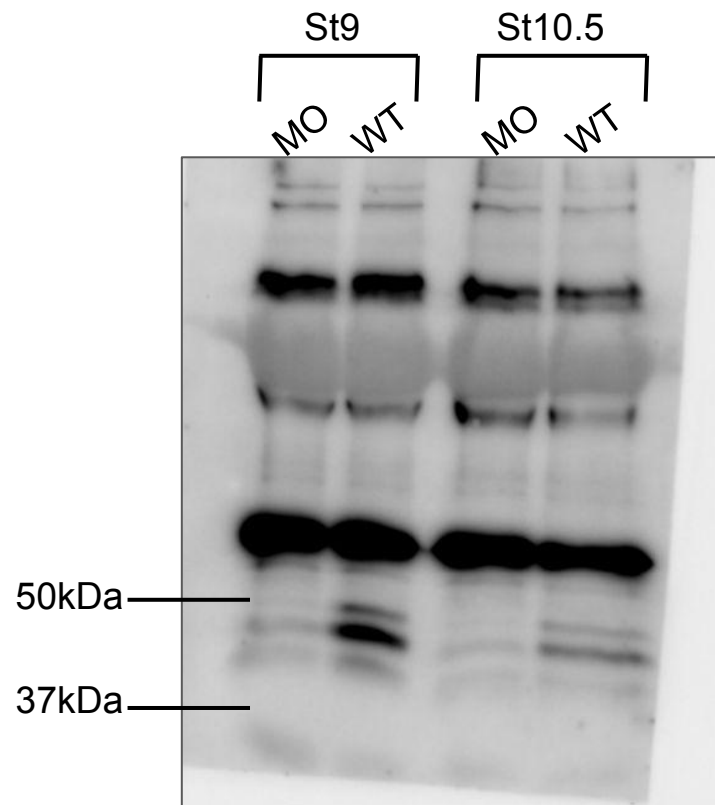

$\alpha$ -Foxi2: IB

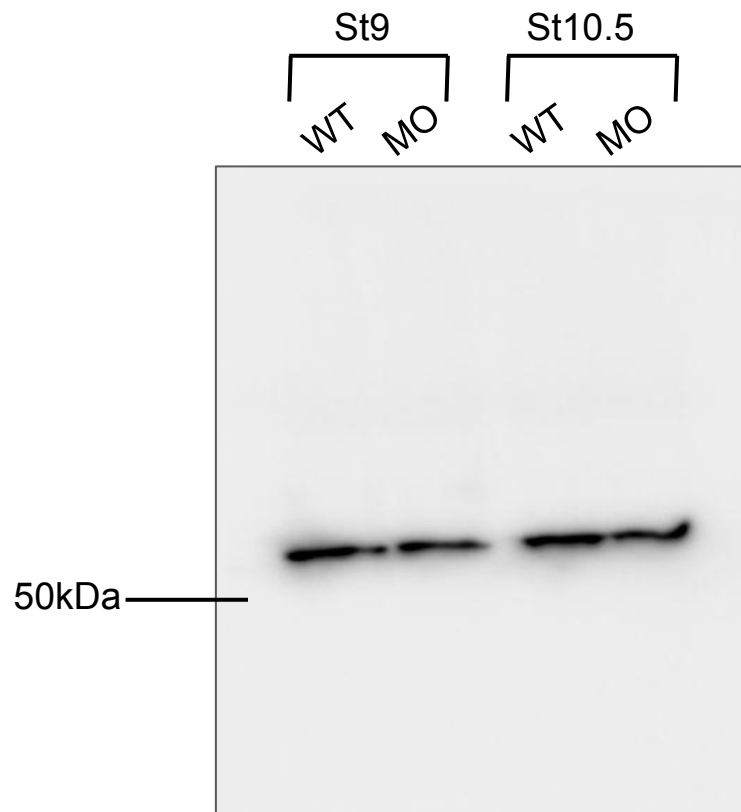

$\alpha$ -Tubulin: IB

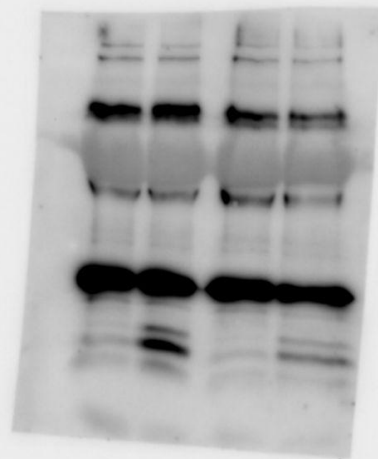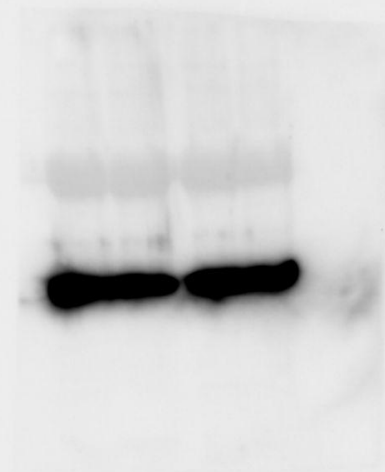

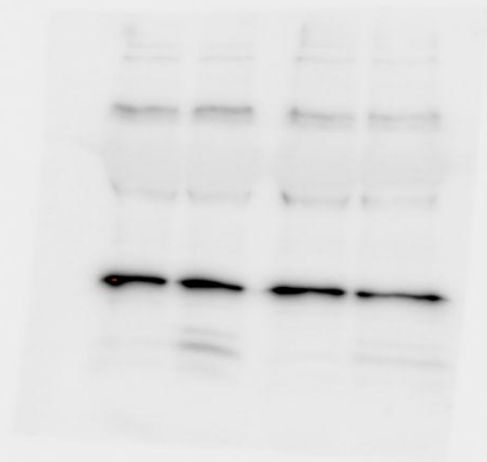

— — — — —

Supplement: S2 Raw Images — Validation of Sox3 Protein Knockdown. Western blot was performed on Stage 9 and 10.5 embryos injected with Sox3 morpholino at the 1–2 cell stage. Sox3 (bottom) and beta-Actin (top) expression are compared between wild-type and morpholino-treated embryos. The Sox3 (bottom) and beta-Actin (top) are the raw exposures used in the main figure text, however, the columns marked “x” were not used to make the publication figure. (PDF) [file pbio.3003476.s020.pdf]
