# Supplementary material for: Foxi2 and Sox3 are master transcription regulators that control ectoderm germ layer specification in Xenopus
Source: PLoS Biol. 2025 Nov 4;23(11):e3003476. doi: 10.1371/journal.pbio.3003476 (PMC12599972; doi:10.1371/journal.pbio.3003476)
Supplement: Attachment — Submitted filename: FoxSoxPaper_ReviewerComments.docx [file pbio.3003476.s021.docx]

**Reviewer 1:**

We would like to thank reviewer #1 for constructive criticisms and have addressed the two major points as follows.

*1. The authors nicely demonstrate that class V-VII genes show increases or constant*

*Foxi2/Sox3 binding during blastula and gastrula and robust transcription. Whereas Class I-III*

*show diminished binding into gastrulation and little gene expression. However, they also claim*

*Foxi2 and Sox3 also pre-bind CRMs for a variety of ectoderm genes at Stage 6.5 (using Chip-*

*qPCR), which is many hours before any of their ChIP-seq data in later blastula. In constast they*

*state that p300 and H3K27ac are not present at this stage. However, as far I can tell they do not*

*provide and ChIP-QPCR data for these factors. It seems possible p300 may be partially*

*enriched at proximal to some genes prior to genome activation in the mid-blastula. Overall I am*

*left wondering the extent to which (1) these maternal factors are pre-bound to germ layer genes*

*prior to genome activation - how many genes, what level of occupancy (compared to in later blastula), (2) whether this early binding is needed for later recruitment of p300, or (3) whether there may be low level recruitment of both the transcription factors and p300, which steadily increase over time during blastula and into gastrulation. In short, is there is a punctuated event, for example after genome activation, and how clearly does binding of TFs precede p300 and histone acetylation. I think the manuscript could be improved by clarifying these points*

We acknowledge the incomplete explanation and agree that the timing of Ep300 recruitment and epigenetic deposition is an important feature of genome activation. Accordingly, we examined Ep300 recruitment to stage 6.5 Foxi2/Sox3-bound CRMs by generating an additional ChIP-qPCR dataset of Ep300 binding (Figure 2G). We demonstrate that while both Foxi2 and Sox3 bind these regions early, Ep300 is not substantially recruited to Foxi2 and Sox3 bound CRMs by stage 6.5. This finding is consistent with previous reports in *Xenopus* indicating that Ep300 is likely first recruited at/after the late blastula stage (stage 9) (Hontelez et al., 2015; Gomes-Faria, 2017, https://discovery.ucl.ac.uk/id/eprint/10047631/). We have also found it difficult to obtain high-quality ChIP-seq results using embryos before stage 8, due to low level of early genomic binding and technical difficulty associated with the limited genomic material in early stage embryos. Scaling up embryo numbers is not a trivial fix as it requires time intensive sonicator recalibration and additional quality control.

To provide stronger direct evidence for a role of Foxi2 and Sox3 in recruiting Ep300, we performed an additional experiment shown in Fig 6D. Specifically, we performed Ep300 ChIP-seq analysis using dissected ectoderm and endoderm explants from Fox/Sox morphants and wild-type embryos. The figure shows that 80% of Ep300 peaks overlapping with Foxi2 and Sox3 are significantly reduced in the absence of these TFs. These experiments demonstrate that both Foxi2 and Sox3 are required for efficient Ep300 recruitment in the genome of ectoderm, but not endoderm by early gastrula stage (Fig. 6D, Fig. Supp. 6B).

Regarding the question of whether Ep300 recruitment is gradual or occurs as a more punctuated event, we are currently unable to address the dynamics of this recruitment. Addressing this question will require the development of new high resolution imaging tools or alternative approaches to temporally inhibit Foxi2 and Sox3 during specific developmental windows.

**Figure 2G**

**Figure 6D**

**Supp. Fig. 6B**

*2. I believe Supp Fig 3 is quite important and I kept referring back to it while reading the paper. This co-binding analysis for Foxi2 and Sox3 feels critical to understanding the manuscript and thus should probably be a main figure (new Figure 4?)*

Thank you for pointing out the usefulness of Supp. Figure 3 for understanding the story. We agree and have accordingly moved Supp. Fig. 3A/B to Figure 2H/I.

**Reviewer 2:**

We would like to thank reviewer 2 for his/her constructive feedback and have addressed both major and minor points individually as follows.

***Main comments:***

*1.) Are most of the regions described in this paper really enhancers or rather promoters given*

*the close proximity to the regulated genes? Perhaps it would be good to state specifically how*

*the authors make that distinction and how they define the term here. Related to this: Is it correct*

*to call Ep300 a marker of enhancers? Or just accessible chromatin?*

The promoter/TSS regions used were default HOMER parameter of (+/-)1000bp from the TSS. Based on this criterion, we find that the vast majority of Foxi2 and Sox3 peaks do not occur within these promoter/TSS regions (see the attached histogram showing the genomic distribution of Foxi2/Sox3 across the genome).

To address whether Ep300 is simply marking open chromatin, we also performed additional analysis to examine the co-occurence of Ep300 peaks and chromatin accessibility as defined by ATAC-seq data. We show in Figure 2D, 3D/E that Ep300 colocalizes with Foxi2 and Sox3, and the nearby genes become actively expressed during gastrulation. These 2,901 Ep300 peaks overlap with DNA accessible sites revealed by ATAC-seq (see figure below left panel), indicating that these regions are, unsurprisingly, in an open chromatin state. In contrast, and more tellingly, the right panel shows that only a fraction of all open chromatin (209,510 ATAC-seq peaks), in the ectoderm contain Foxi2, Sox3 and Ep300 binding. Together, these data indicate that Foxi2 and Sox3 preferentially bind to specific accessible regions that are also enriched for Ep300, rather than binding indiscriminately across all open chromatin regions.

*2.) Why does it make sense that repressive marks on ectodermal genes are missing in*

*endoderm? This is quite interesting, but not much discussed by the authors.*

We do agree that this is interesting, however we refrained from much speculation because we don’t believe there is enough data to generate a sensible model. However, we have added a sentence to the text noting that our dissected ChIP-seq data are consistent with observations in Akkers et al., 2009 (page 6, ln 143-145).

*3.) Fig 3 A/B: class II and IV peaks are co occupied by Foxi2 and Sox3, but are excluded from*

*analysis in Fig3D because low number of uniquely bound genes. This is confusing to me.*

*Please clarify.*

In Figure 3A classes II and III have only 110 and 180 peaks, respectively, corresponding to 5 and 64 associated genes. We therefore removed the data from the original figure. However, to avoid confusion we have added them back into the figure, along with class IV (Figure 2D, see below).

**Figure 2D**

*4.) Fig 4: Was this a mild phenotype or why does it make sense that ectodermal structures are*

*still generated after MO against foxi2 and sox3? In sox3 morphants only 5 of 20 are having the*

*phenotype. This makes me wonder about the robustness of the phenotype and why the*

*embryos is shown as repressive?*

Morpholino knockdowns of Foxi2 and Sox3 are efficient in blocking expression of these TFs but the ectodermal germ layer isn’t completely obliterated by loss of these factors. It’s clear that there is more to ectodermal specification than Foxi2 and Sox3 alone, but these lie at the top of a network, along with other factors, in regulating ectodermal gene activation at ZGA. Blocking expression does have phenotypic effects on anterior structures and overall morphology of the embryos. We acknowledge and state in the text (p 9, ln 269-270) that this phenotype is observed in a minority of Sox3 morphants.

*5.) One of the main implicated consequences of their model is that Foxi2 and Sox3 are required*

*for Ep300 to bind specific regions in the ectoderm to modify histones required for active gene*

*expression. However, a definitive experiment addressing that is missing. Perhaps adding an*

*experiment where Ep300 ChIP is performed after Foxi2 and Sox3 knockdown in ectoderm vs*

*endoderm explants would be quite elucidating here. Ep300 binding should be lost in the*

*ectoderm, but not in the endoderm.*

We agree with reviewer 2’s point and accordingly generated new Ep300 ChIP-seq datasets using the ectoderm and endoderm of Foxi2/Sox3 (FS) morphants and control embryos. These data show a significant reduction of Ep300 recruitment at most of FSP-bound regions in the ectoderm (Figure 6D), with no statistically notable change in Ep300 recruitment at regions that lack FS binding, nor at Ep300 peaks associated with endodermally-expressed genes (Supp. Fig. 6B).

**Figure 6D**

**Supp. Fig. 6B**

**Minor comments:**

*1.) Fig 1 SC color code needs clarification. No size of region indicated.*

Thank you for your note. Supp. Fig. 1C has been updated (see below).

*2.) Fig 2E: there is no rational given as to why it could make sense that foxi2 peaks coincide*

*with sox/fox motifs but not fox/pou motifs. Would be nice to discuss/explain a bit more.*

While we acknowledge the high degree of Fox and Sox motif co-occurrence in Foxi1/Ep300 peaks, we also would like to point out that the quantification of Fox/Pou motif co-occurrence in Figure 2F demonstrates that ~40% of Foxi2/Ep300 peaks contain both Fox and Pou motifs. While co-occurrence with a Pou motif is lower than the frequency of having a Sox motif, we do find that the Fox/Pou motifs do co-occur appreciably. We now more explicitly discuss the presence of Pou motifs (page 7, ln 197-200).

*3.) Clarification on ChIP analysis: please state in the text that antibodies were used against*

*endogenous proteins/markers and how these were validated to act specific. Also, the methods*

*state that chip peaks were detected using input data from 2017. So no input data was generated*

*with the presented Foxi2, Sox3, histone and Ep300 ChIP? This seems a bit odd. How do the*

*authors make sure that technical artifacts are the same across studies/methods/reagents?*

All antibodies used have been previously validated to recognize the respective endogenous proteins or histone modifications. The Foxi2 antibody was validated in Cha et al., 2012, Sox3 antibody was validated in Zhang et al., 2004, the Ep300 antibody is widely used in many systems for ChIP-seq and was validated for use in *Xenopus* in Hontelez et al. 2015, the H3K27ac antibody is widely used and was validated for use in *Xenopus* in Gupta et al., 2014, and Charney et al., 2017, the H3K4me1 antibody is widely used and was validated for use in *Xenopus* in Gupta et al., 2014, Hontelez et al., 2015; and Paraiso et al., 2019, 2025.

Regarding the ChIP-seq input data, we present the following analysis to reviewer #2. The table below shows the Foxi2 and Sox3 ChIP-seq data analyses using different input datasets from animal cap, marginal zone, vegetal pole and whole embryo at the equivalent stage. Comparing Foxi2 ChIP-seq peak calls from this study and other input controls from animal cap, marginal zone, vegetal pole and whole embryo samples shows 97.5%, 96.5%, 96.6%, and 96.5% peak call overlaps, respectively (see table below). Similarly, comparing the overlap between Sox3 ChIPseq peak calls from this paper and the same additional inputs yields 93.9%, 93.6%, 93.1%, 93.2% of peak overlap, respectively (see table below). These analyses demonstrate that input datasets are highly reproducible across experiments and yield very similar peak calling. These datasets were used for germ layer specific peak calling in the manuscript but we neglected to add them to methods and are now added and in GEO (page 23, ln 741).

*4.) It would be nice to discuss the hand over to other TFs and how that might affect sub-*

*lineages in the ectoderm a bit more, however only when space permits.*

In the discussion section titled, “The roles of maternal Foxi2 and Sox3 in early development” we briefly touch on the notion of maternal-zygotic TF hand-offs. However, we did not discuss its potential role in sub-lineage commitment. Our bulk RNAseq analysis reveals that Foxi2 and Sox3 each regulate both distinct and shared targets, and our gastrula single-nucleus RNA-seq analysis reveals that these targets are expressed in specific ectoderm sub-lineages. The finding suggests lineage-specific hand-offs to specific zygotic TFs. Unfortunately, at present we cannot speculate as to which ectoderm sub-lineage cells have accessible Foxi2/Sox3/Ep300 CRMs to identify potential hand-off partners. A multiomic dataset would allow us to identify lineage specific CRMs and examine for enriched motifs corresponding to candidate zygotic TFs.
