# Supplementary material for: Foxi2 and Sox3 are master transcription regulators that control ectoderm germ layer specification in Xenopus
Source: PLoS Biol. 2025 Nov 4;23(11):e3003476. doi: 10.1371/journal.pbio.3003476 (PMC12599972; doi:10.1371/journal.pbio.3003476)
Supplement: Attachment — Submitted filename: Response- Editorial Questions .docx [file pbio.3003476.s022.docx]

***Responses to Editorial Questions***

*We would like you to consider a suggestion to improve the title:
"Foxi2 and Sox3 are master transcription regulators that control ectoderm germ layer specification in Xenopus"*

We have modified the title and running title of the paper as suggested.

*As you address these items, please take this last chance to review your reference list to ensure that it is complete and correct. If you have cited papers that have been retracted, please include the rationale for doing so in the manuscript text, or remove these references and replace them with relevant current references. Any changes to the reference list should be mentioned in the cover letter that accompanies your revised manuscript.*

A new reference has been added to provide proper credit for the drawing used in the figures.

Zahn N, James-Zorn C, Ponferrada VG, Adams DS, Grzymkowski J, Buchholz DR, Nascone-Yoder NM, Horb M, Moody SA, Vize PD, Zorn AM. 2022. Normal Table of *Xenopus* development: a new graphical resource. Development, 149 (14): dev200356.

**Formatting changes:**

1) *State the initials, alongside each funding source, of each author to receive each grant.*

We have modified the funding source section.

2) *We have noticed that you have uploaded Supporting Information files, but you have not included a complete list of legends. Please add a full list of legends for your Supporting Information files (Sox3_WB.pdf) and (Foxi2_WB.pdf) after the references list*.

Legends for Supporting Information files, Sox_WB.pdf and Foxi2_WB.pdf have been included after the main and supplemental figure legend (p40).

3*) Potential Copyright Issues: Figures 4, 6a, and S4a: Please confirm whether you drew the images / clip-art within the figure panels by hand. If you did not draw the images, please provide (a) a link to the source of the images or icons and their license / terms of use; or (b) written permission from the copyright holder to publish the images or icons under our CC BY 4.0 license. Alternatively, you may replace the images with open source alternatives.*

Regarding Figures 4, 6a, and S4a, we now cite *Xenopus* illustrations © Natalya Zahn (2022) in figure legend and in reference

Zahn N, James-Zorn C, Ponferrada VG, Adams DS, Grzymkowski J, Buchholz DR, Nascone-Yoder NM, Horb M, Moody SA, Vize PD, Zorn AM. 2022. Normal Table of *Xenopus* development: a new graphical resource. Development, 149 (14): dev200356.).

We have also modified Figure 5C and added our own figure.

*2) Deposition in a publicly available repository. Please also provide the accession code or a reviewer link so that we may view your data before publication.*

GSE288636, GSE288637, GSE288638, GSE303467

*Regardless of the method selected, please ensure that you provide the individual numerical values that underlie the summary data displayed in the following figure panels as they are essential for readers to assess your analysis and to reproduce it:Fig. 1A, C; Fig. 2B-G; Fig. 3B-E; Fig. 4C-E; Fig. 5B; Fig. 6A-E, G-I; Fig. S2A, B; Fig. S3A, C; Fig. S4A, B; Fig. S5A and Fig. S6B-G*

We now have provided requested information.

*Please also ensure that figure legends in your manuscript include information on WHERE THE UNDERLYING DATA CAN BE FOUND, and ensure your supplemental data file/s has a legend.*

The updated figure legends now include information on where the data underlying each figure can be found.

*Please also make the data you have deposited in GEO publicly available at this stage.*

All data deposited in GEO are now publicly available.
